# Triglyceride and glucose (TyG) index as a predictor of incident hypertension: a 9-year longitudinal population-based study

**DOI:** 10.1186/s12944-017-0562-y

**Published:** 2017-09-13

**Authors:** Rongjiong Zheng, Yushan Mao

**Affiliations:** 1 Department of Endocrinology, the Affiliated Hospital of Ningbo University School of Medicine, 247 Renmin Road, Ningbo, 315020 China; 2 Ningbo University, Ningbo, China

**Keywords:** Triglyceride and glucose index, Hypertension, Epidemiology

## Abstract

**Background:**

Hypertension and the triglyceride and glucose index both have been associated with insulin resistance; however, the longitudinal association remains unclear. This study was designed to investigate the longitudinal association between the triglyceride and glucose index and incident hypertension among the Chinese population.

**Methods:**

We studied 4686 subjects (3177 males and 1509 females) and followed up for 9 years. The subjects were divided into four groups based on the triglyceride and glucose index. Univariate and multivariate Cox regression models were used to analyse the risk factors of hypertension.

**Results:**

After 9 years of follow-up, 2047 subjects developed hypertension. The overall 9-year cumulative incidence of hypertension was 43.7%, ranging from 28.5% in quartile 1 to 36.9% in quartile 2, 49.2% in quartile 3 and 59.8% in quartile 4 (*p* for trend < 0.001). Cox regression analyses indicated that higher triglyceride and glucose index was associated with an increased risk of subsequent incident hypertension.

**Conclusion:**

The triglyceride and glucose index can predict the incident hypertension among the Chinese population.

## Background

With the population aging, hypertension (HTN) has become the most important risk factor for cardiovascular disease (CVD) morbidity and mortality [1–4]. According to a WHO report, it was estimated that the HTN mortality was about 55% worldwide in 2013 [5, 6]. Considering that HTN has been one of the most serious public health issues [7], an effective and accurate predictor for the incident HTN should be needed nowadays.

Recently, the triglyceride and glucose (TyG) index has been proposed as a simple surrogate of insulin resistance (IR) [8, 9], and numerous studies have also found a relationship between HTN and IR [10, 11]. Additionally, a recent cross-sectional study revealed that the TyG index is closely associated with HTN [12]; however, the longitudinal association remains unclear. Therefore, we performed a 9-year longitudinal population-based study to investigate the association between the TyG index and incident HTN among the Chinese population.

## Methods

### Study population

The study participants, aged 20–80 years, were enrolled in the annual physical health examinations at Zhenhai Lianhua Hospital in Ningbo, China. This longitudinal population-based cohort study was conducted to investigate the longitudinal association between the TyG index and the incident HTN beginning from 2006 to 2015. The subjects were excluded according to the following criteria: (1). Subjects who had a history of HTN or overt cardiovascular diseases. (2). Subjects who were taking medicines that may affect blood pressure or lipids. (3). Subjects who were drinking alcohol and smoking at study entry. In total, 4686 subjects including 3177 males and 1509 females were evaluated for the study.

### Survey and measurements

1.  A questionnaire was completed for the initial health examinations in 2006 at the health check-up center of Zhenhai Lianhua Hospital through face-to-face interviews by a well-trained senior physician. The questionnaire included the subjects’ demographic characteristics, smoking and drinking status, medical history, and family background.
2.  Anthropometric test: Standing height and body weight was measured while he/she was in light indoor clothing without shoes. Body mass index (BMI) was calculated as weight in kilograms divided by height in meters squared. Waist circumference (WC) was measured at the level midway between the lower rib margin and the iliac crest [13]. Sitting blood pressure was measured from the right arm three times with a 1-min interval between the measurements after the rest for 20 min by trained members, using an automated device (Omron HEM-7052; Omron Corp., Kyoto, Japan). The mean value of the three measurements was calculated for analysis.
3.  Laboratory examinations: Venous blood samples were obtained from the subjects in the morning after at least 12 h prior to the examination. Routine biochemical data including blood urea nitrogen (BUN), creatinine (Cr), triglyceride (TG), total cholesterol (TC), high density lipoprotein cholesterol (HDL-C), low density lipoprotein (LDL-C), serum uric acid (SUA), fasting plasma glucose (FPG), alanine aminotransferase (ALT), aspartate aminotransferase (AST), γ-glutamyltransferase (γ-GGT), Apo-A1and Apo-B were estimated using an Olympus AU640 automated analyser (Olympus, Kobe, Japan). All of the laboratories involved resoundingly completed the standardization.

### Definitions

1.  HTN was defined as systolic blood pressure (SBP) ≥ 140 mmHg, diastolic blood pressure (DBP) ≥ 90 mmHg or current drug use for HTN in accordance with the criteria of the WHO [14].
2.  The TyG index was calculated with established formulas according to the previous studies [15, 16]: TyG = Ln [TG (mg/ml) * FPG (mg/ml) /2]
3.  The estimated glomerular filtration rate (eGFR) was calculated using the improved Chinese population Modification of Diet in Renal Disease (MDRD) study formula [17].

### Statistical analysis

The fundamental characteristics of the samples were summarized by descriptive statistics. All statistical analyses were performed using SPSS software (version 17.0, SPSS software, Chicago, IL, USA). Continuous variables were expressed as median (IQR) and categorical variables were presented as percentages (%). Continuous variables were compared using the student’s t text, Mann-Whitney *U* test, Kruskal-Wallis *H* test or one-way ANOVA depending on the normality of the data. Categorical variables between groups were compared using Chi-square text. For a statistical inference, all *p* values are bilateral, and a *p* value of less than 0.05 was considered statistically significant.

The subjects were classified into four groups (quartile 1 to quartile 4) based on the TyG index at baseline. The classifications of the TyG index in these groups were as follows: quartile 1(Q1) (≤ 8.12), quartile 2(Q2) (8.13–8.46), quartile 3(Q3) (8.47–8.83), and quartile 4(Q4) (≥ 8.84) for male; and quartile 1(Q1) (≤ 7.85), quartile 2(Q2) (7.86–8.16), quartile 3(Q3) (8.17–8.52), and quartile 4(Q4) (≥ 8.53) for female. The basic characteristics of the subjects in each group were compared. The cumulative incidence of HTN was calculated by dividing the number of cases by the numbers of subjects followed up for each TyG group. Cox proportional hazards regression models were used to analyze the risk of incident HTN for each baseline TyG group.

## Results

### Basic characteristics

The basic demographic and clinical characteristics of the subjects are presented in Table 1. In the study, a total of 4686 subjects including 3177 males and 1509 females were evaluated at baseline. Significant differences in age, BMI, SBP, DBP, WC, TyG, FPG, UA, AST, ALT, γ-GGT, TC, TG, LDL-C, HDL-C, Apo-B and eGFR between the groups were observed (*p* < 0.001). In addition, age, BMI, SBP, DBP, WC, FPG, UA, AST, ALT, γ-GGT, TC, TG, LDL-C and Apo-B all tended to increase in subjects with a higher TyG index, whereas HDL-C and eGFR were significantly lower in subjects with a higher TyG index (*p* < 0.001).

**Table 1:** Baseline characteristics of the subjects according to TyG quartiles

Variables	TyG quartile				
	Quartile 1 (*n* = 1149)	Quartile 2 (*n* = 1183)	Quartile 3 (*n* = 1184)	Quartile 4 (*n* = 1170)	*p*
TyG	7.81 (7.65–7.97)	8.21 (8.09–8.33)	8.55 (8.42–8.68)	9.04 (8.88–9.30)	< 0.001
Gender (male/%)	783/68.1	801/67.7	799/67.5	794/67.9	0.99
Age (years)	37.0 (32.0–44.0)	39.0 (34.0–48.0)	41.0 (35.0–51.0)	45.0 (37.0–54.0)	< 0.001
BMI (kg/m^2^)	21.0 (19.6–22.9)	21.9 (20.2–24.0)	23.0 (21.2–24.7)	23.9 (22.2–25.7)	< 0.001
SBP (mmHg)	113.0 (106.0–122.0)	115.0 (107.0–124.0)	119.0 (111.0–127.0)	122.0 (114.0–128.0)	< 0.001
DBP (mmHg)	72.0 (67.0–78.0)	74.0 (68.0–79.0)	76.0 (70.0–81.0)	78.0 (73.0–83.0)	< 0.001
WC (cm)	73.0 (68.0–79.0)	76.0 (70.0–82.0)	79.0 (72.0–85.0)	82.0 (76.0–88.0)	< 0.001
BUN (μmol/L)	4.97 (4.24–5.75)	4.93 (4.25–5.76)	5.02 (4.18–5.82)	4.97 (4.16–5.82)	0.894
Cr (μmol/L)	72.0 (61.0–81.0)	73.0 (62.0–81.0)	73.0 (61.0–82.0)	72.0 (61.0–81.0)	0.849
FPG (mmol/L)	4.25 (3.99–4.51)	4.39 (4.12–4.67)	4.48 (4.20–4.81)	4.67 (4.34–5.09)	< 0.001
UA (μmol/L)	305.0 (251.0–352.0)	311.0 (253.0–367.0)	333.0 (269.0–392.0)	351.0 (286.0–402.0)	< 0.001
AST (U/L)	18.0 (16.0–22.0)	19.0 (16.0–23.0)	20.0 (17.0–24.0)	21.0 (18.0–26.0)	< 0.001
ALT (U/L)	18.0 (14.0–25.0)	20.0 (15.0–30.0)	24.0 (17.0–36.0)	28.0 (20.0–42.0)	< 0.001
γ-GGT (U/L)	14.0 (11.0–20.0)	16.0 (12.0–23.0)	19.0 (13.0–31.0)	25.0 (16.0–41.0)	< 0.001
TC (mmol/L)	4.28 (3.78–4.80)	4.58 (4.07–5.11)	4.78 (4.21–5.35)	5.12 (4.54–5.78)	< 0.001
TG (mmol/L)	0.73 (0.61–0.85)	1.05 (0.90–1.20)	1.44 (1.21–1.67)	2.28 (1.87–2.92)	< 0.001
HDL-C (mmol/L)	1.36 (1.11–1.67)	1.29 (1.08–1.59)	1.22 (1.05–1.50)	1.22 (1.071.45)	< 0.001
LDL-C (mmol/L)	2.27 (1.90–2.68)	2.56 (2.10–3.05)	2.73 (2.26–3.26)	2.89 (2.39–3.46)	< 0.001
Apo-A1(g/L)	1.33 (1.17–1.49)	1.31 (1.14–1.49)	1.28 (1.11–1.49)	1.27 (1.10–1.47)	0.228
Apo-B(g/L)	0.77 (0.64–0.90)	0.86 (0.73–1.00)	0.95 (0.81–1.10)	1.06 (0.89–1.23)	< 0.001
eGFR (mL/(min·1.73 m^2^))	112.2 (99.8–127.1)	109.9 (99.1–123.4)	109.0 (97.1–123.7)	108.9 (96.3–123.5)	< 0.001

### Association between the TyG index and incident HTN

**Fig. 1:**
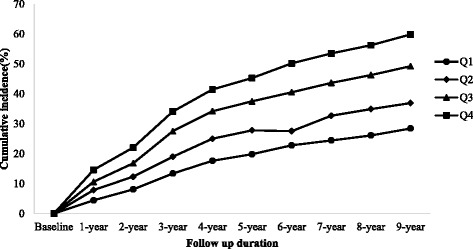
The association between the baseline TyG index and the cumulative incidence of HTN

To investigate the predictive value of the TyG index for incident HTN, our longitudinal study was conducted for 9 years. After a 9-year follow-up, 2047 subjects (1541 males and 506 females) developed HTN, corresponding to 48.5 and 33.5% cumulative incidence of HTN in males and females, respectively. Figure 1 shows that the baseline TyG index predicted the incidence of HTN in a positive and dose-responsive manner. The overall 9-year cumulative incidence of HTN was 43.7%, ranging from 28.5% in quartile 1 to 36.9% in quartile 2, 49.2% in quartile 3 and 59.8% in quartile 4 (*p* for trend < 0.001; Fig. 1). This tendency also holds true for 1- to 8-year cumulative incidences. These findings indicate that those with higher TyG groups are more likely to develop HTN. In addition, the subjects with incident HTN are predominantly male, and the baseline age, BMI, SBP, DBP, WC, TyG, BUN, Cr, FPG, UA, AST, ALT, γ-GGT, TC, TG, LDL-C, and Apo-B in subjects with incident HTN were significantly higher than the other group (Table 2).

**Table 2:** Baseline characteristics of the subjects according to follow-up outcomes

Variables	Subjects developed HTN (*n* = 2047)	Subjects did not develop HTN (*n* = 2639)	*p*
TyG	8.55 (8.20–8.92)	8.23 (7.92–8.59)	< 0.001
Gender (male/female, n)	1541/506	1636/1003	< 0.001
Age (years)	44.0 (36.0–54.0)	38.0 (33.0–46.0)	< 0.001
BMI (kg/m^2^)	23.6 (21.8–25.4)	21.7 (19.8–23.6)	< 0.001
SBP (mmHg)	124.0 (117.0–131.0)	112.0 (105.0–120.0)	< 0.001
DBP (mmHg)	80.0 (75.0–84.0)	71.0 (67.0–76.0)	< 0.001
WC (cm)	81.0 (75.0–87.0)	75.0 (69.0–81.0)	< 0.001
BUN (μmol/L)	5.09 (4.29–5.96)	4.86 (4.15–5.66)	< 0.001
Cr (μmol/L)	74.0 (64.0–83.0)	71.0 (60.0–80.0)	< 0.001
FPG (mmol/L)	4.53 (4.21–4.89)	4.37 (4.09–4.66)	< 0.001
UA (μmol/L)	342.0 (284.0–395.0)	306.0 (250.0–364.0)	< 0.001
AST (U/L)	20.0 (17.0–25.0)	19.0 (16.0–23.0)	< 0.001
ALT (U/L)	25.0 (17.0–37.0)	20.0 (15.0–30.0)	< 0.001
γ-GGT (U/L)	21.0 (15.0–34.0)	16.0 (11.0–24.0)	< 0.001
TC (mmol/L)	4.83 (4.28–5.50)	4.56 (3.99–5.14)	< 0.001
TG (mmol/L)	1.38 (1.01–2.02)	1.07 (0.80–1.51)	< 0.001
HDL-C (mmol/L)	1.26 (1.07–1.52)	1.27 (1.08–1.58)	0.020
LDL-C (mmol/L)	2.73 (2.24–3.29)	2.51 (2.04–3.03)	< 0.001
Apo-A1 (g/L)	1.29 (1.13–1.50)	1.30 (1.13–1.48)	0.815
Apo-B (g/L)	0.97 (0.81–1.14)	0.85 (0.71–1.01)	< 0.001
eGFR (mL/(min·1.73 m^2^))	108.0 (95.0–122.0)	111.6 (100.2–126.2)	< 0.001

### The TyG index and the risk of incident HTN

We used univariate and multivariate Cox proportional hazard models to analyse the hazard ratio for incident HTN in each TyG group in our study (Tables 3 and 4). Compared with the lowest TyG group, the hazard ratios (95% CI) for subjects in quartile 2, quartile 3 and quartile 4 were 1.38 (1.20–1.59), 2.00 (1.74–2.29) and 2.63 (2.31–3.00), respectively (*p* for trend < 0.001). The same relationship between the TyG index and incident HTN was also revealed even after adjusting for age and gender (Mode 1); or age, gender, and BMI (Mode 2); or age, gender, and all clinical variables (Mode3) in Table 4. These findings indicate that a higher TyG index is associated with an increased risk of subsequent incident HTN.

**Table 3:** Univariate and multivariate Cox Proportional Hazard models of development of HTN during 9-year follow-up

Variables	Univariate models		Multivariate models	
	HR (95%CI)	*P* value	HR (95%CI)	*p* value
Gender (male)	1.62 (1.46–1.78)	< 0.001	1.33 (1.14–1.56)	< 0.001
Age (years)	1.03 (1.03–1.04)	< 0.001	1.03 (1.02–1.03)	< 0.001
BMI (kg/m^2^)	1.16 (1.15–1.18)	< 0.001	1.09 (1.06–1.12)	< 0.001
WC (cm)	1.06 (1.05–1.06)	< 0.001	1.01 (1.00–1.02)	0.017
BUN (mmol/L)	1.11 (1.07–1.15)	< 0.001	1.00 (0.96–1.04)	0.967
Cr (μmol/L)	1.00 (1.00–1.01)	< 0.001	1.00 (1.00–1.01)	0.018
FPG (mmol/L)	1.26 (1.21–1.30)	< 0.001	1.10 (1.03–1.18)	0.006
UA (μmol/L)	1.01 (1.00–1.00)	< 0.001	1.00 (1.00–1.00)	0.006
AST (U/L)	1.01 (1.00–1.01)	< 0.001	1.00 (1.00–1.01)	0.648
ALT (U/L)	1.00 (1.00–1.00)	< 0.001	1.00 (1.00–1.00)	0.433
γ-GGT (U/L)	1.00 (1.00–1.01)	< 0.001	1.00 (1.00–1.00)	0.001
TC (mmol/L)	1.28 (1.22–1.34)	< 0.001	1.16 (0.95–1.43)	0.144
TG (mmol/L)	1.29 (1.25–1.33)	< 0.001	1.08 (0.94–1.24)	0.264
HDL-C (mmol/L)	0.86 (0.77–0.97)	0.010	0.96 (0.74–1.25)	0.756
LDL-C (mmol/L)	1.31 (1.24–1.38)	< 0.001	0.84 (0.70–1.02)	0.073
Apo-A1 (g/L)	1.02 (0.86–1.21)	0.812	1.07 (0.86–1.33)	0.562
Apo-B (g/L)	3.47 (2.95–4.09)	< 0.001	1.15 (0.78–1.70)	0.490
eGFR (mL/(min·1.73 m^2^))	1.00 (0.99–1.00)	< 0.001	1.00 (1.00–1.00)	0.238
TyG		< 0.001		0.013
Quartile 1	1.00 (reference)		1.00 (reference)	
Quartile 2	1.38 (1.20–1.59)		1.21 (1.00–1.47)	
Quartile 3	2.00 (1.74–2.29)		1.49 (1.16–1.93)	
Quartile 4	2.63 (2.31–3.00)		1.53 (1.07–2.19)

**Table 4:** Risk of development HTN according to baseline TyG categories in unadjusted and adjusted models

Models	Quartile 1 (n = 1149)	Quartile 2 (n = 1183)	Quartile 3 (n = 1184)	Quartile 4 (n = 1170)	*p*
Unadjusted	1.00 (reference)	1.38 (1.20–1.59)	2.00 (1.74–2.29)	2.63 (2.31–3.00)	< 0.001
Mode 1 (Adjusted for age and gender)	1.00 (reference)	1.32 (1.14–1.52)	1.79 (1.56–2.05)	2.21 (1.94–2.53)	< 0.001
Mode 2 (Adjusted for age, gender, and BMI)	1.00 (reference)	1.19 (1.03–1.38)	1.51 (1.32–1.74)	1.71 (1.48–1.96)	< 0.001
Mode 3 (Adjusted for age gender and all clinical variables^a^)	1.00 (reference)	1.21 (1.00–1.47)	1.49 (1.16–1.93)	1.53 (1.07–2.19)	0.013

^a^Including BMI, WC, BUN, Cr, FPG, UA, AST, ALT, γ-GGT, TC, TG, HDL-C, LDL-C, Apo-A1, Apo-B, and eGFR

## Discussion

This population-based study demonstrates that there is a longitudinal relationship between the TyG index and the risk of incident HTN during a 9-year period among the Chinese population. We also found that the TyG index is an independent predictor for incident HTN. Cox regression analysis suggests that subjects with a higher baseline TyG index are significantly associated with a higher risk of incident HTN after adjusting for the confounders. This study also confirms that the findings of relevant cross-sectional studies, which observe an independent positive relationship between the TyG index and incident HTN [12].

IR has been the significant risk factor for the development of HTN [10, 18] and it may be the mechanism in developing HTN in the population. Firstly, hyperinsulinemia caused by IR may increase sympathetic nervous system activity, promote the secretion of adrenaline and norepinephrine, and finally increase cardiac output and peripheral vascular resistance [19]. The high concentration of catecholamine may thicken the vascular smooth muscle, and lead to luminal stenosis or HTN [20]. Secondly, IR may also increase the activity of the renin-angiotensin-aldosterone system (RAAS), promote the reabsorption of H_2_O and Na^+^ indirectly, cause water-sodium retention and increase the vascular activity with noradrenaline and AT-II, leading to the HTN eventually [21, 22]. Slater [23] also found that IR could promote sodium retention in the kidneys in an animal experiment. Thirdly, IR could also increase the synthesis and release of endothelin, which may contract the blood vessels, and decrease the synthesis of prostacyclin (PGI2) and prostaglandin E2 (PGE2), which may dilate vessels [24, 25], and finally induce the proliferation of the vascular smooth muscle, resulting in blood pressure elevation.

Previous epidemiological studies have shown that the TyG index was a well-known predictor for the development of diabetes mellitus [26]. Our results indicated that the TyG index predicted the subsequent occurrence of HTN in a positive and dose-dependent manner. Therefore, the early detection of the TyG index may be beneficial for early interventions to prevent HTN among the Chinese population.

The major strengths of this study were the 9-year longitudinal population-based study and the large number of the subjects. This longitudinal study also expanded the observation to establish the temporal sequence between the TyG index and the later risk of HTN in China. Moreover, the selection bias was less likely to appear in the present study as annual health check-ups in state-owned companies are mandatory in China. However, certain limitations also exist in this study. First, fasting insulin was not obtained due to the lack of relevant devices. Second, we did not record the information on nutritional habits or energy intake in this study. Although we did not adjust for this possible confounding factor, we used other additional covariates indirectly related to nutritional habits, such as BMI or cholesterol. Third, taking lipid-lowering medications or nutraceuticals might have influenced the lipids or the TyG index values. However, the percentage of subjects on these agents was towards the null, we did not think that it would influence the results. Fourth, physical activity may affect the blood pressure or lipids. However, the duration and intensity of our subjects’ physical activity in this study was almost similar, we did not think it would affect the results either. Finally, all subjects in the present study were enrolled from one hospital, so the conclusions may differ from the general population. Given these limitations, further studies should be carried out to clarify the above factors.

## Conclusion

In conclusion, our study showed that the TyG index independently predicted the incident HTN. These findings suggested that the TyG index should be introduced to the routine check-ups, which could benefit for the prevention of HTN.

## Abbreviations

ALT: Alanine aminotransferase
AST: Aspartate aminotransferase
BMI: Body mass index
BUN: Blood urea nitrogen
Cr: Creatinine
CVD: Cardiovascular disease
DBP: Diastolic blood pressure
eGFR: Estimated glomerular filtration rate
FPG: Fasting plasma glucose
HDL-C: High density lipoprotein cholesterol
HTN: Hypertension
IR: Insulin resistance
LDL-C: Low density lipoprotein
RAAS: Renin-angiotensin-aldosterone system
SBP: Systolic blood pressure
SUA: Serum uric acid
TC: Total cholesterol
TG: Triglyceride
TyG: Triglyceride and glucose
WC: Waist circumference
γ-GGT: γ-glutamyltransferase
